# Association between dyslipidaemia and the risk of hyperuricaemia: a six-year longitudinal cohort study of elderly individuals in China

**DOI:** 10.1080/07853890.2022.2118368

**Published:** 2022-09-02

**Authors:** Ying Xu, Haoyu Dong, Boya Zhang, Jiayu Zhang, Qinghua Ma, Hongpeng Sun

**Affiliations:** a School of Public Health, Medical College of Soochow University, Suzhou, China; b The 3rd People’s Hospital of Xiangcheng District, Suzhou, China; c Jiangsu Key Laboratory of Preventive and Translational Medicine for Geriatric Diseases, Medical College of Soochow University, Suzhou, China

**Keywords:** Dyslipidaemia, hyperuricaemia, longitudinal cohort study, stratification analyses, dose-response relationship

## Abstract

**Background:**

Despite abundant evidence linking dyslipidaemia to an increased risk of hyperuricaemia, the exact association between each component of dyslipidaemia and hyperuricaemia remains controversial. Thus, the objective of this research was to examine the correlation between dyslipidaemia and its components, as well as hyperuricaemia in Chinese people over the age of 60.

**Methods:**

In this study, 4018 participants over 60 years without hyperuricaemia were investigated from 2014 to 2020. The association between each dyslipidaemia component and hyperuricaemia was evaluated employing Cox proportional hazards models. This research conducted further stratified and sensitivity analyses to assess the potential relationship.

**Results:**

A total of 1155 participants suffered from hyperuricaemia (28.75%) at the time of the 6-year follow-up survey. In multivariable-adjusted analyses, compared to participants with normal lipid levels, those with dyslipidaemia had 1.28 times the risk (95% confidence interval 1.12 to 1.47) of experiencing hyperuricaemia. The hazard ratios (HR) (95% CI) comparing high TC, high TG, high LDL-C, and low HDL-C of dyslipidaemia with the regular group were 0.99 (0.72 to 1.37), 1.30 (1.07 to 1.57), 1.02 (0.70 to 1.50), and 1.20 (1.00 to 1.44), respectively. There was a nonlinear dose-response between TG, HDL-C, and serum uric acid (SUA).

**Conclusions:**

Dyslipidaemia and its two distinct types, high TG and low HDL-C, increased hyperuricaemia incidence in this prospective cohort. Further research should be undertaken to investigate the possible reverse causality between different components of dyslipidaemia and hyperuricaemia.

## Background

Hyperuricaemia (HUA) is a metabolic condition characterised by high serum uric acid (SUA) levels or decreased uric acid excretion in the body as a result of purine metabolism problems. Because of its high incidence and the elevated risk of several non-communicable diseases (NCDs) such as dyslipidaemia, hypertension, and cardiovascular disease [1–3], HUA has become a significant public health problem worldwide_._ Its prevalence is approximately 20% worldwide [4]. As many NCDs are age-related, older people have become one of the most vulnerable groups to these diseases. The World Health Organisation defines older people as those over 60 years of age. It is estimated that China’s elderly population will exceed 350 million [5]. However, as life expectancy increases, the burden of NCDs will also increase. In China, the overall prevalence of HUA in older people >60 years of age was 13.1%, at a relatively high level [6]. Patients with HUA are more likely to be affected by adverse health outcomes. With the rapid ageing of the population, there is an urgent need for measures to reduce the risk of NCDs in older people, which would further benefit global sustainable development and the healthy ageing process.

High total cholesterol (TC), high triglyceride (TG), low-density lipoprotein cholesterol (LDL-C), or high-density lipoprotein cholesterol (HDL-C) levels are the components of dyslipidemia, which is a disorder caused by faulty lipid metabolism and excessive or inadequate lipoprotein synthesis in the plasma [7]. Considerable evidence has proved that dyslipidaemia is significantly associated with elevated SUA levels or HUA [8–10]. However, the correlation between components of dyslipidaemia and HUA remains controversial. Some studies have found that TG levels, but not HDL-C levels, were significantly closely correlated with HUA [11,12], whereas other studies demonstrated that HDL-C was inversely related to SUA levels [13]. Several cross-sectional studies have reported that dyslipidaemia may be associated with the development of HUA, hampering causal inferences [14–16]. Furthermore, few studies have assessed the relevance of individual components of dyslipidemia to HUA in Chinese older adults. Consequently, scientific research should be undertaken to explore the relationship between various dyslipidaemias and HUA.

This work aimed to see if there was a link between each component of dyslipidaemia and HUA. Therefore, this study performed a prospective cohort study based on elderly individuals older than 60 with a six-year follow-up from 2014 to 2020.

## Methods

### Setting and participants

**Figure 1.**
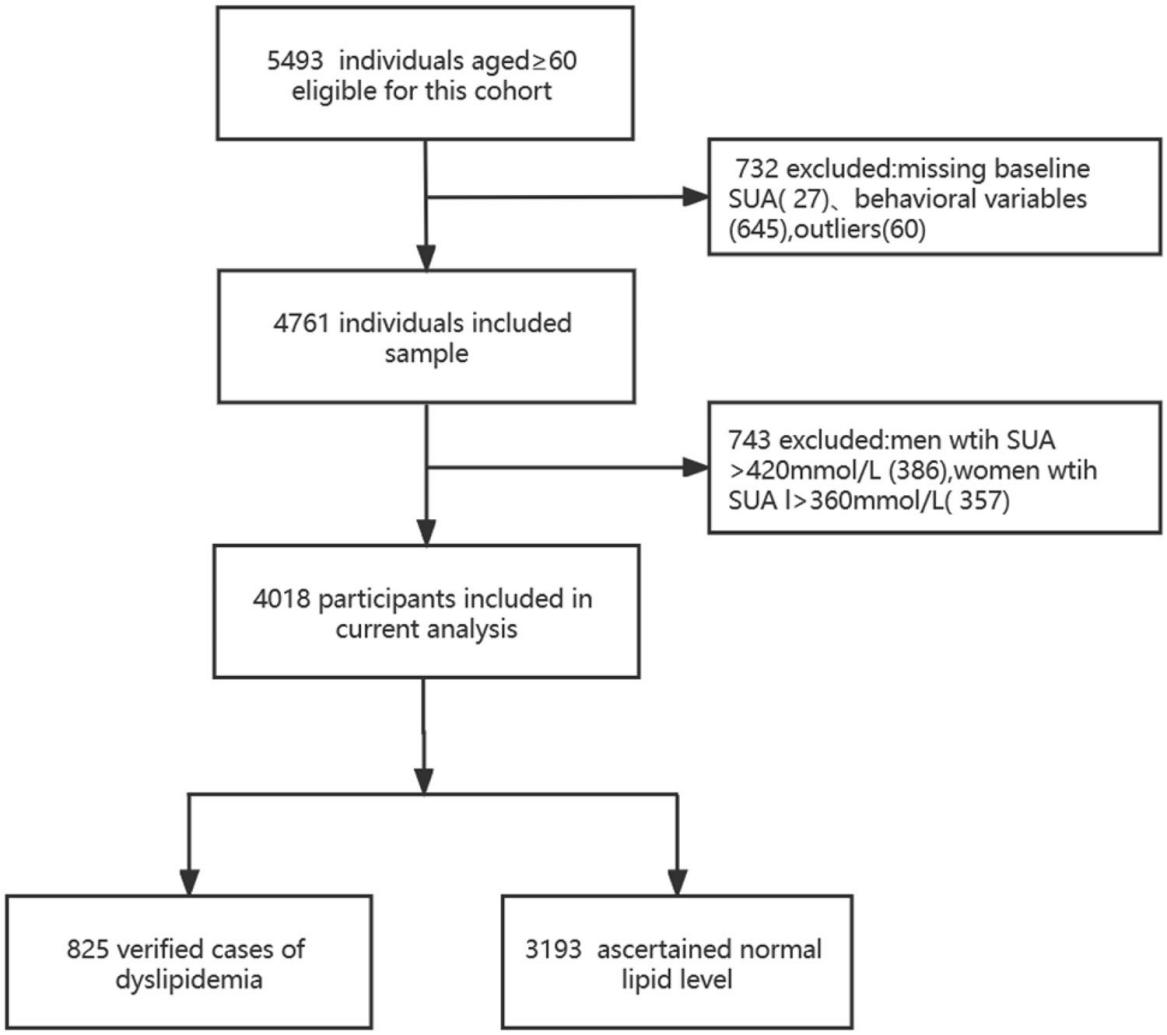
Flow chart of subject selection for the present study.

Data were derived from the Weitang Geriatric Diseases Study, which is a community-based survey conducted in Weitang Town located in Suzhou, a metropolis in East China. At the start of the trial in 2014, 5493 participants aged 60 and up were enrolled. From this cohort, it excluded 27 subjects with missing baseline serum uric acid values, 645 subjects who had missing health behaviour variables, 60 subjects with outliers, and 734 subjects with HUA at baseline, leaving 4018 participants who were free of HUA to take part in the study between 2014 and 2020. The details of the participants’ selection process are presented in Figure 1. The Weitang Geriatric Diseases Study was funded by the National Natural Science Foundation of China in 2014 (81402761) and approved by the Institutional Review Board of Soochow University. All participants received written informed consent.

Based on their initial blood lipid levels, participants with aberrant lipids were split into an exposure group and subjects with normal lipids into a control group. Of 844 people with dyslipidaemia, 71 had high TG, 155 had high TC, 22 had high LDL-C, 292 had low HDL-C, and 304 suffered from more than one dyslipidaemia component.

### Data collection

All eligible persons were encouraged to take part in the study. Sociodemographic variables and health-related behaviour variables were collected using a standard questionnaire. Sociodemographic data included age and gender. Variables affecting health behaviour were smoking status (yes/no), alcohol consumption (yes/no), and physical activity (yes/no). Body measurements included body mass index (BMI), systolic blood pressure (SBP), and diastolic blood pressure (DBP). SUA, TC, TG, HDL-C, LDL-C, creatinine (CRE), blood urea nitrogen (BUN), fasting blood glucose (FPG), alanine aminotransferase (ALT), and aspartate aminotransferase (AST) levels were all determined through laboratory tests.

### Definitions

HUA was classified as SUA ≥ 420 mmol/L (≥7.0 mg/dL) in men and ≥360 mmol/L (6.0 mg/dL) in women [17]. Participants were considered to have dyslipidaemia if they met at least one of the following criteria at the baseline examination: (1) TC ≥ 6.22 mmol/L (≥240 mg/dL); (2) TG ≥ 2.26 mmol/L (≥ 200 mg/dL); (3) LDL-*C* ≥ 4.14 mmol/L (≥ 160, mg/dL); and (4) HDL-*C* ≤ 1.04 mmol/L (40 mg/dL) [7]. Diabetes was commonly defined as FPG ≥ 7.0 mmol/L, the use of anti-diabetic medications, or a diagnosis of diabetes mellitus [18]. Participants were diagnosed with hypertension if their SBP was ≥ 140 mmHg and/or DBP ≥90 mmHg or a previous history of hypertension [19]. BMI was calculated by dividing weight by height (kg/m^2^). BMI ≥ 25 kg/m^2^ was defined as overweight according to World Health Organisation criteria.

### Statistical analyses

The mean ± standard deviation (SD) describes continuous variables, while percentages describe categorical variables. The unpaired Student’s t-test and Chi-square tests were used to compare the individual characteristics of the two groups.

Cox proportional hazards regression models were further constructed to calibrate the hazard ratios (HRs) and 95% confidence intervals (CIs) of incident HUA events to evaluate the association between dyslipidaemia and HUA after attempting to control for covariates by stepwise adjustments in three models: Model 1 was modified for age and gender; Model 2 was comparable to Model 1, but included smoking status, alcohol intake, physical activity, hypertension, diabetes, and levels of CRE, BUN, ALT, and AST; Model 3 was similar to Model 2, but with additional adjustments for BMI, TC, TG, HDL-C, and LDL-C values. The dose-response connection between each dyslipidaemia component and HUA was visually quantified using a regression model with limited cubic splines.

Further stratified analyses, which depended on age (60–69 years or ≥70 years), sex (female or male), BMI (<25 or ≥25), and hypertension (yes or no), were performed to evaluate potential confounding factors that could have an impact on the correlation between dyslipidaemia and HUA.

To minimise potential bias, this study also performed sensitivity analyses that excluded participants who were confirmed as having HUA within two years of follow-up or those with baseline cardiovascular disease, hypertension, or diabetes.

STATA 15.0 and SAS 9.4 were used to conduct all statistical analyses, with a statistical significance level of *p* < .05 (two-sided).

## Results

### Baseline characteristics

This study recruited 4018 medically certified individuals with an average age of 67.70 ± 6.43 years. 1895 males (47.16%) with a mean age of 67.63 ± 6.23 years and 2123 women (52.84%) with an average age of 67.76 ± 6.61 years participated in the work. By the end of the research, there were 1155 new cases of HUA, with a 6-year cumulative incidence of 28.75%. This study divided all participants into two groups based on their lipid levels: the dyslipidaemia group and the regular lipid group. BMI, SBP, DBP, FPG, and ALT levels, alcohol intake, history of hypertension, and history of diabetes were significantly higher among those with dyslipidaemia (Table 1). Other characteristics such as age, gender, smoking status, physical activity, AST level, and CRE level were not substantially different between the two groups.

**Table 1.** Baseline characteristics of subjects participants by the group of blood lipid.

Characteristics	Dyslipidaemia (*n* = 844)	Non-Dyslipidaemia (*n* = 3174)	*p* Value
Age, years, mean ± SD	67.30 ± 6.10	67.80 ± 6.52	.12
Gender, n (%)			.21
man	382 (45.26)	1513 (47.67)	
woman	462 (54.74)	1661 (52.33)	
BMI, kg/m^2^	24.31 ± 3.27	22.77 ± 3.06	<.01
Smoking status, n (%)			.81
yes	291 (34.48)	1080 (34.03)	
no	553 (65.52)	2094 (65.97)	
Alcohol intake, n (%)			<.01
yes	145 (17.18)	718 (22.62)	
no	699 (82.82)	2456 (77.38)	
Physical activity, n (%)			.35
yes	368 (43.60)	1327 (41.81)	
no	476 (56.40)	1847 (58.19)	
SBP, mmHg	146.22 ± 19.80	142.72 ± 19.00	<.01
DBP, mmHg	86.57 ± 11.19	85.13 ± 11.20	<.01
FPG, mmol/L	5.74 ± 1.31	5.52 ± 1.16	<.01
ALT, U/L	21.30 ± 13.56	18.33 ± 10.82	<.01
AST, U/L	24.14 ± 10.05	24.06 ± 8.07	.05
Creatinine,μmol/L	68.24 ± 14.79	67.73 ± 15.12	.23
BUN, mmol/L	4.94 ± 1.27	5.29 ± 1.42	<.01
Hypertension, n (%)			<.01
yes	569 (67.42)	1936 (61.00)	
no	275 (32.58)	1238 (39.00)	
Diabetes, n (%)			<.01
yes	93 (11.02)	206 (6.49）	
no	751 (88.98)	2968 (93.51)	

### Associations between the components of dyslipidaemia and HUA

Table 2 demonstrates the association between the components of dyslipidaemia and HUA in all groups by multivariate Cox proportional hazard regression analyses. HRs and 95% CIs of HUA to dyslipidaemia and each of its components were determined. After the total adjustment for potential confounders in Model 3, dyslipidaemia and its components of high TG and low HDL-C levels remained significantly correlated with HUA, with HRs of 1.28 (1.12 to 1.47), 1.30 (1.07 to 1.57) and 1.20 (1.00 to 1.44), respectively. The study observed no significant associations between high TC, high LDL-C, and HUA in any of the models.

**Table 2.** Hazard ratio for the incidence of HUA by dyslipidaemia and its components.

	Normal group	Exposure group	For each SD
**Dyslipidaemia**			
Incident rate 10000 person-years	1840.74/10000	1981.61/10000	–
model1^a^	1.0 (ref)	1.52 (1.33–1.73)	–
model2^b^	1.0 (ref)	1.40 (1.23–1.60)	–
model3^c^	1.0 (ref)	1.28 (1.12–1.47)	–
**High TC**			
Incident rate 10000 person-years	2001.89/10000	2019.54/10000	–
model1^a^	1.0 (ref)	1.18 (0.94–1.47)	1.06 (1.00–1.14)
model2^b^	1.0 (ref)	1.06 (0.84–1.32)	1.06 (0.99–1.16)
model3^c1^	1.0 (ref)	0.99 (0.72–1.37)	0.88 (0.64–1.19)
**High TG**			
Incident rate 10000 person-years	2004.03/10000	1992.06/10000	–
model1^a^	1.0 (ref)	1.66 (1.39–1.98)	1.24 (1.17–1.31)
model2^b^	1.0 (ref)	1.48 (1.24–1.78)	1.18 (1.12–1.26)
model3^c2^	1.0 (ref)	1.30 (1.07–1.57)	1.15 (1.00–1.32)
**High LDL-C**			
Incident rate 10000 person-years	2003.00/10000	2002.92/10000	–
model1^a^	1.0 (ref)	1.17 (0.90–1.53)	1.14 (1.05–1.23)
model2^b^	1.0 (ref)	1.07 (0.82–1.40)	1.05 (0.96–1.15)
model3^c3^	1.0 (ref)	1.02 (0.70–1.50)	1.15 (0.76–1.73)
**Low HDL-C**			
Incident rate 10000 person-years	1851.33/10000	1945.95/10000	–
model1^a^	1.0 (ref)	1.52 (1.29–1.79)	0.56 (0.48–0.66)
model2^b^	1.0 (ref)	1.44 (1.22–1.71)	0.59 (0.50–0.70)
model3^c4^	1.0 (ref)	1.20 (1.00–1.44)	0.84 (0.60–1.19)

model 1^a^: adjusted for age and gender;.

model 2^b^: model1^a^+ smoking status, alcohol intake, physical activity, hypertension, diabetes, CRE, BUN, AST, and ALT.

model 3^c^: model 2^b^+BMI; model 3^c1^: model 3^c^+TG, LDL-C, and HDL-C; model 3^c2^: model 3^c^+ TC, LDL-C, and HDL-C; model 3^c3^: model 3^c^+TG, TC, and HDL-C; model 3^c4^: model 3^c^+TG, TC, and LDL-C.

**Figure 2.**
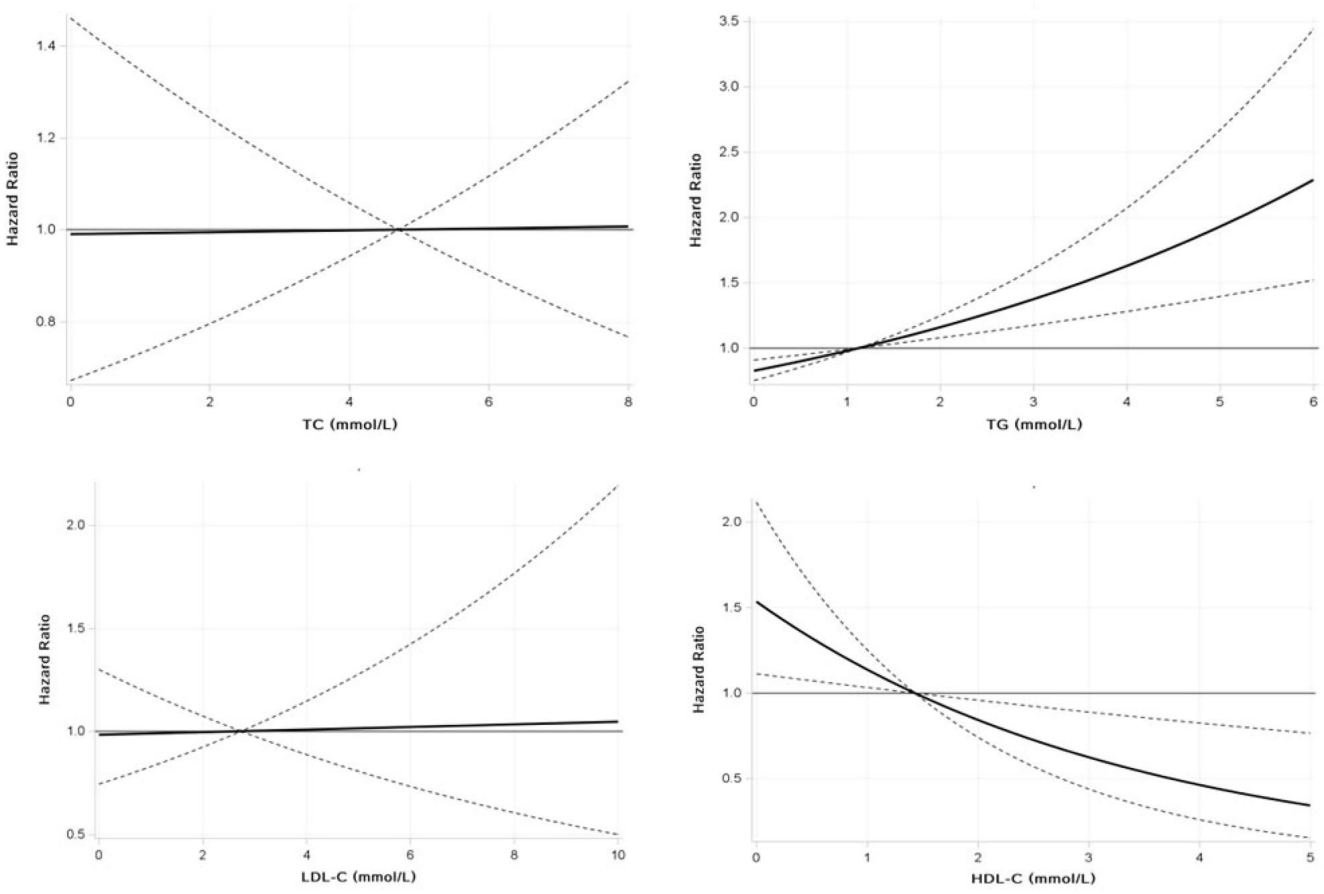
Risk of incident HUA associated with different dyslipidaemia types during the 6 years.

This research found evidence of nonlinear associations for TG levels, with a strong association with HUA at higher concentrations but a weaker association at low to moderate concentrations, and HDL-C levels, with a strong inverse association with HUA at low to moderate concentrations but a weaker association at higher concentrations (Figure 2).

Table 3 presents the association between the increasing number of dyslipidaemia components and HUA. The HRs for HUA were statistically significant for one, two, or more components of each cumulative number compared with reference values for non-dyslipidaemia components: the HRs were 1.27 (1.08 to 1.49) and 1.30 (1.07 to 1.58), respectively.

**Table 3.** Multivariable Cox regression analysis of between number of dyslipidaemia components and HUA.

Number of dyslipidaemia components	Case(n)	Model 1	Model 2	Model 3
0	3174	1(ref)	1(ref)	1(ref)
1	540	1.49 (1.28–1.75)	1.40(1.21–1.65)	1.27 (1.08–1.49)
2 and more	304	1.54 (1.27–1.87)	1.39 (1.14–1.69)	1.30 (1.07–1.58)

Model 1: adjusted for age and gender.

Model 2: adjusted for age, gender, smoking status, alcohol intake, physical activity, hypertension, diabetes, CRE, BUN, AST, and ALT.

Model 3: adjusted for age, gender, smoking status, alcohol intake, physical activity, hypertension, diabetes, CRE, BUN, AST, ALT, and BMI.

The solid black line and the dashed area represent estimates of HRs and the 95% CIs, respectively, for each dyslipidaemia type. Covariates included age, gender, BMI, smoking status, alcohol intake, physical activity, hypertension, diabetes, CRE, BUN, AST, and ALT.

### Subgroup analyses

**Figure 3.**
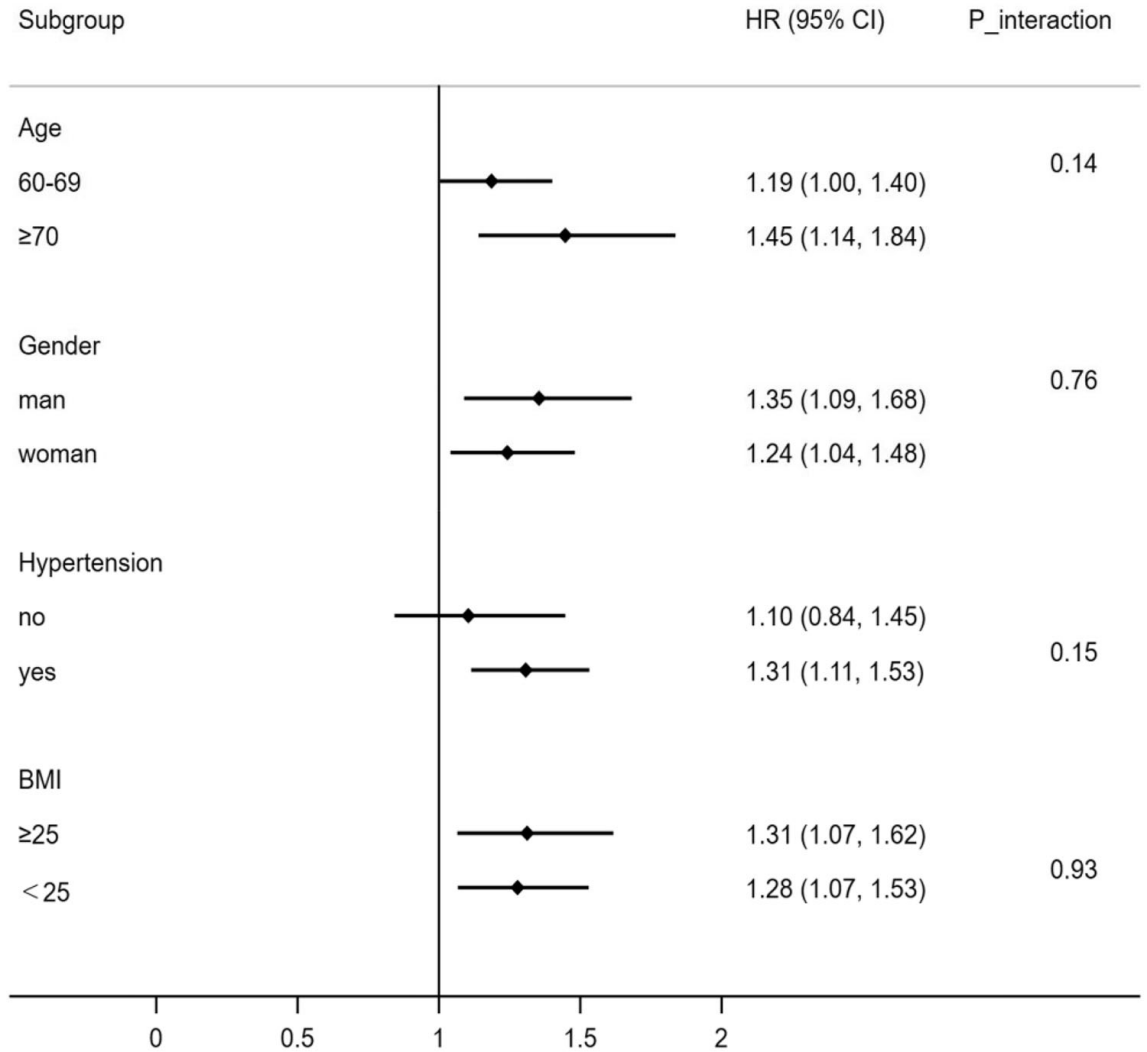
Stratified analyses for the association between dyslipidaemia and HUA.

The research employed stratified analyses to see if age, gender, BMI level, and hypertension had any effect on the connection between dyslipidaemia and HUA. As shown in Figure 3, after controlling for possible confounding factors, the stratification analyses revealed that the association between dyslipidaemia and HUA was prominent in individuals aged 70 years and older (HRs1.45, 95% CIs1.14 to 1.84), men (1.35, 1.09 to 1.68), individuals with a BMI ≥ 25 (1.31,1.07 to 1.62) and people with hypertension (1.31, 1.11 to 1.52). However, dyslipidaemia did not significantly interact with any of the variables mentioned above.

When analysing a subgroup variable, age, gender, BMI, diabetes, and hypertension were all adjusted except the variable itself.

### Sensitivity analyses

In sensitivity analyses, after mutual adjustment, the association between dyslipidaemia and HUA was attenuated after excluding the first two years of incident HUA or baseline diabetes or cardiovascular cases (Table 4). When this study excluded participants who developed hypertension events, dyslipidaemia was no longer significantly associated with HUA.

**Table 4.** Sensitivity analyses for the association between dyslipidaemia and HUA.

Model	Number	HRs (95% CIs)	
		Non-dyslipidaemia	Dyslipidaemia
Model 3, excluding the first 2 years’ incident HUA	3694	1.0 (ref)	1.30 (1.11–1.52)
Model 3, excluding baseline cardiovascular cases	3728	1.0 (ref)	1.29 (1.12–1.48)
Model 3, excluding baseline hypertension cases	1513	1.0 (ref)	1.12 (0.86–1.45)
Model 3, excluding baseline diabetes cases	3719	1.0 (ref)	1.29 (1.12–1.48)

Model 3: adjusted for age, gender, smoking status, alcohol intake, physical activity, hypertension, diabetes, CRE, BUN, AST, ALT, and BMI.

## Discussion

Even after correcting for putative confounding variables, dyslipidaemia, high TG, and low HDL-C are significantly and independently related to the risk of developing HUA in this 6-year prospective analysis of elderly persons aged more than 60 years living in China. Furthermore, the dose-response association between TG, HDL-C, and SUA demonstrated a nonlinear increasing trend. Moreover, the link between an expanding number of dyslipidaemia components and HUA seemed somewhat stronger in mixed dyslipidaemia participants. These findings have increased epidemiological evidence of an association between various dyslipidaemia components and HUA.

## Comparisons with other studies

Many studies have confirmed that dyslipidaemia may cause an increase in HUA. However, whether all dyslipidaemia components are involved in HUA is still debateable. The positive association between TG levels and HUA risk is supported by previous studies [11,20,21]. Nakanishi et al. [22] found that basal TG remained an independent predictor of new-onset HUA even after excluding patients with diabetes mellitus and those on long-term medications for certain chronic conditions, which was consistent with our study. This study shows that dyslipidaemia increases the risk of HUA and that the risk varies by clinical subtype, with the highest risk in those with high TG. The specific mechanism of elevated TG levels and HUA has not been elaborated. One potential explanation is that increased free fatty acid production and utilisation will be induced in the body with the increase in TG levels, and the catabolism of adenosine triphosphate will be accelerated, causing an increase in SUA production [23]. Scholars have concluded that there are differences in the link between HDL-C levels and HUA. Abbas Dehghan *et al.* [24] concluded that there was no correlation between HDL-C levels and SUA. However, the present research discovered a nonlinear dose-response trend of increasing the incidence of HUA with decreasing HDL-C levels, implying that a low HDL-C level can trigger HUA. HDL-C has anti-inflammatory, antioxidant, and anti-apoptotic effects [25]. Some preliminary research has found that HDL-C reduces urate crystallization-induced inflammation, indicating that HDL is involved in the uric acid-induced inflammatory response [26].

In this study, increased dyslipidaemic components are positively associated with HUA. The findings of this research highlight that epidemiological data lays the groundwork for future research into determining the association between components of dyslipidaemia and HUA.

In a population aged 70 years and older, dyslipidaemia was associated with higher hazard ratios for HUA than in people younger than 70 years. Because the activity of various chemical enzymes involved in the body’s metabolic process becomes abnormal with increasing age, the movement of some of the enzymes involved in the process of purine metabolism increases accordingly, giving rise to the cumulative concentration of blood uric acid in the elderly population. Therefore, priority attention should be given to the older age group with dyslipidaemia.

With respect to sex differences, it is well known that males have higher uric acid levels than females [27,28]. Several earlier studies have concluded that the link between dyslipidaemia and HUA in women is not substantial [29]. In a gender-stratified analysis, however, the study discovered this link in male and female subjects, with the association appearing to be more pronounced in males. This phenomenon is mainly related to biological differences [30]. Androgens can promote the reabsorption of blood uric acid in the body and have a critical influence in hindering the excretion of blood uric acid [31]. In contrast, oestrogen plays a protective role for women, inhibiting the reabsorption of uric acid by the kidneys. In addition, chronic poor lifestyles such as smoking, excessive alcohol consumption, and irregular diet in men lead to more exogenous purines entering the body. The result is a considerable accumulation of lactic acid in the body during metabolism, which prevents the normal excretion of uric acid in the blood, thus triggering an increase in blood uric acid concentration. This behaviour pattern gradually raises the male population’s blood uric acid level.

A considerable amount of literature has been published showing that obesity and hypertension are closely related to dyslipidaemia and HUA [2,32,33]_._ Previous studies have shown that insulin resistance or HUA is pivotal in the correlation between dyslipidaemia and the risk of HUA. One possible explanation is that insulin resistance partially overlaps with the pathophysiological features of hypertension, obesity, dyslipidaemia, and HUA, as higher insulin levels reduce renal uric acid excretion [34–36]. A longitudinal study of nondiabetic Japanese men showed that insulin resistance or hyperinsulinaemia (increased TG concentrations and decreased HDL-C concentrations) might lead to the incidence of HUA, which is similar to our study [37].

## Clinical and public health potential

Different lipid components and SUA were discovered to have a dose-response relationship in this investigation. The elderly population at risk of HUA should have their lipid levels actively managed. The focus should not only be on correcting HUA at the expense of lipid levels, nor should it only be on the dyslipidaemia that increases the risk of HUA. Blood uric acid and lipid levels in older people should be tested in tandem to take comprehensive and effective measures to improve their health and quality of life.

It is recommended that targeted health management should be carried out for different genders and age groups of older people. That appropriate treatment guideline should be developed through dietary, behavioural, lifestyle, and pharmacological control measures to prevent elevated lipid levels, thereby reducing the adverse effects of lipids on the incidence of HUA and improving the health of the region’s older population. For example, a cross-sectional study of adults aged 18 to 79 in rural China found that an increase in healthy lifestyle factors significantly improved lipid profiles [38]. According to a survey, reducing the amount of animal- and soy-based meals consumed helps minimise the risk of HUA [39].

In particular, to prevent HUA development, special emphasis should be paid in clinical work to the early diagnosis and management of persons with high lipid levels, particularly those with high TG and low HDL-C.

## Strengths and limitations of the present study

The strength of the research lies in the prospective study with a long follow-up period. The discovery of a link between dyslipidaemia and HUA established a solid theoretical foundation for HUA prevention and treatment. In addition, this study analysed lipids as both categorical and continuous variables, avoiding data loss and allowing researchers to dig deeper into the underlying relationship between multiple dyslipidaemic components and HUA. Furthermore, this study performed a subgroup and sensitivity analysis of the risk of various dyslipidaemia and HUA after correcting for numerous possible confounders.

Despite these strengths, the present work has several limitations. First, the sample size of participants with physical examination data was not large enough to satisfy the completeness of the survey. Second, this study only measured the relevant indices at 2-time points of baseline follow-up and did not analyse the impact of dynamic changes in the index in the follow-up results. Thirdly, although adjustments were made for a range of potential confounders, the possibility of residual and unmeasured confounders, such as information on diet, medications, and genetics, could not be excluded. These factors may have different effects on the risk of HUA. Relevant data were not collected for this study. Finally, this study was based on a community-based study in China, and therefore the observed associations were somewhat limited when extrapolated to other regions. The results await further validation in a multicentre, long-term prospective cohort study.

## Conclusions

In conclusion, this cohort investigation demonstrated that the dyslipidaemia components of high TG and low HDL-C positively correlate with the incidence of HUA in the elderly population. Targeted health management of the elderly with dyslipidaemia is recommended, with particular attention to early diagnosis and management for those with excessive lipid levels to avoid the development of HUA. Enhanced detection of dyslipidaemia and HUA in the elderly can contribute to developing and progressing chronic disease complications. Further research should be undertaken to investigate the possible reverse causality between different components of dyslipidaemia and HUA.

## Data Availability

All the data and materials used in our article are available from the corresponding author on reasonable request.

## Abbreviations

HUA: hyperuricaemia
SUA: serum uric acid
TC: total cholesterol
TG: triglycerides
LDL-C: low-density lipoprotein cholesterol
HDL-C: high-density lipoprotein cholesterol
BMI: body mass index
SBP: systolic blood pressure
DBP: diastolic blood pressure
FPG: fasting plasma glucose
CRE: creatinine
BUN: blood urea nitrogen
ALT: alanine aminotransferase
AST: aspartate transaminase
HR: hazard ratio
CI: confidence interval
SD: standard deviation

## References

[1] Filiopoulos V, Hadjiyannakos D, Vlassopoulos D. New insights into uric acid effects on the progression and prognosis of chronic kidney disease. Ren Fail. 2012;34(4):510–520. 22260409 10.3109/0886022X.2011.653753

[2] Wei FJ, Sun N, Cai CY, et al. Associations between serum uric acid and the incidence of hypertension: a chinese senior dynamic cohort study. J Transl Med. 2016;14(1):110. 27129957 10.1186/s12967-016-0866-0 PMC4851787

[3] Kim SY, Guevara JP, Kim KM, et al. Hyperuricemia and coronary heart disease: a systematic review and Meta-Analysis. Arthritis Care Res. 2010;62(2):170–180. 10.1002/acr.20065 PMC3156692 20191515

[4] Zhu YY, Pandya BJ, Choi HK. Prevalence of gout and hyperuricemia in the US general population the national health and nutrition examination survey 2007-2008. Arthritis Rheum. 2011;63(10):3136–3141. 21800283 10.1002/art.30520

[5] Zhang J, Song PK, Zhao LY, et al. Malnutrition in relation with dietary, geographical, and socioeconomic factors among older chinese. Biomed Environ Sci. 2021;34(5):337–347. 34059170 10.3967/bes2021.045

[6] Wang R, Tang Z, Sun F, et al. Prevalence of hyperuricemia in the elderly in 7 areas of China. Zhonghua Liu Xing Bing Xue Za Zhi. 2018;39(3):286–288. 29609240 10.3760/cma.j.issn.0254-6450.2018.03.007

[7] Jacobson TA, Maki KC, Orringer CE, NLA Expert Panel, et al. National lipid association recommendations for Patient-Centered management of dyslipidemia: Part 2. J Clin Lipidol. 2015;9(6 Suppl):S1–122.e1. 10.1016/j.jacl.2015.09.002 26699442

[8] Kuwabara M, Borghi C, Cicero AFG, et al. Elevated serum uric acid increases risks for developing high LDL cholesterol and hypertriglyceridemia: a five-year cohort study in Japan. Int J Cardiol. 2018;261:183–188. 29551256 10.1016/j.ijcard.2018.03.045

[9] Liang J, Jiang YT, Huang YF, et al. The comparison of dyslipidemia and serum uric acid in patients with gout and asymptomatic hyperuricemia: a cross-sectional study. Lipids Health Dis. 2020;19(1):31. 32127000 10.1186/s12944-020-1197-y PMC7053114

[10] Chen SH, Yang H, Chen YS, et al. Association between serum uric acid levels and dyslipidemia in chinese adults a cross-sectional study and further Meta-analysis. Medicine. 2020;99(11):e19088. 32176036 10.1097/MD.0000000000019088 PMC7440131

[11] Zhang Y, Wei FJ, Chen C, et al. Higher triglyceride level predicts hyperuricemia: a prospective study of 6-year follow-up. J Clin Lipidol. 2018;12(1):185–192. 29137896 10.1016/j.jacl.2017.10.009

[12] Goncalves JP, Oliveira A, Severo M, et al. Cross-sectional and longitudinal associations between serum uric acid and metabolic syndrome. Endocrine. 2012;41(3):450–457. 22350659 10.1007/s12020-012-9629-8

[13] Son MK, Seo J, Yang S. Association between dyslipidemia and serum uric acid levels in korean adults: Korea national health and nutrition examination survey 2016-2017. PLoS ONE. 2020;15(2):e0228684. 32059030 10.1371/journal.pone.0228684 PMC7021293

[14] Ni Q, Lu XM, Chen CC, et al. Risk factors for the development of hyperuricemia a STROBE-compliant cross-sectional and longitudinal study. Medicine (Baltimore). 2019;98(42):e17597. 31626136 10.1097/MD.0000000000017597 PMC6824661

[15] Peng TC, Wang CC, Kao TW, et al. Relationship between hyperuricemia and lipid profiles in US adults. Biomed Res Int. 2015;2015:1–7. 10.1155/2015/127596 PMC4299312 25629033

[16] Ali N, Rahman S, Islam S, et al. The relationship between serum uric acid and lipid profile in bangladeshi adults. BMC Cardiovasc Disor. 2019;19:42. 10.1186/s12872-019-1026-2 PMC6385393 30791868

[17] Fang J, Alderman MH. Serum uric acid and cardiovascular mortality the NHANES I epidemiologic follow-up study, 1971-1992. National health and nutrition examination survey. JAMA. 2000;283(18):2404–2410. 10815083 10.1001/jama.283.18.2404

[18] Jia WP, Weng JP, Zhu DL, Chinese Diabetes Society, et al. Standards of medical care for type 2 diabetes in China. 2019Diabetes Metab Res Rev. 2019;35(6):e3158. 30908791 10.1002/dmrr.3158

[19] Wang ZW, Chen Z, Zhang LF, On behalf of the China Hypertension Survey Investigators*, et al. Status of hypertension in China results from the China hypertension survey, 2012-2015. Circulation. 2018;137(22):2344–2356. 29449338 10.1161/CIRCULATIONAHA.117.032380

[20] Lu WL, Song K, Wang Y, et al. Relationship between serum uric acid and metabolic syndrome: an analysis by structural equation modeling. J Clin Lipidol. 2012;6(2):159–167. 22385549 10.1016/j.jacl.2011.11.006

[21] Stelmach MJ, Wasilewska N, Wicklund-Liland LI, et al. Blood lipid profile and BMI-Z-score in adolescents with hyperuricemia. Ir J Med Sci. 2015;184(2):463–468. 24876094 10.1007/s11845-014-1146-8

[22] Nakanishi N, Tatara K, Nakamura K, et al. Risk factors for the incidence of hyperuricaemia: a 6-year longitudinal study of Middle-aged japanese men. Int J Epidemiol. 1999;28(5):888–893. 10597987 10.1093/ije/28.5.888

[23] Balasubramanian T. Uric acid or 1-methyl uric acid in the urinary bladder increases serum glucose, insulin, true triglyceride, and total cholesterol levels in wistar rats. ScientificWorldJournal. 2003;3:930–936. 15241498 10.1100/tsw.2003.90 PMC5974611

[24] Dehghan A, van Hoek M, Sijbrands EJG, et al. High serum uric acid as a novel risk factor for type 2 diabetes. Diabetes Care. 2008;31(2):361–362. 17977935 10.2337/dc07-1276

[25] Nicholls SJ, Nelson AJ. HDL and cardiovascular disease. Pathology. 2019;51(2):142–147. 30612759 10.1016/j.pathol.2018.10.017

[26] Scanu A, Luisetto R, Oliviero F, et al. High-density lipoproteins inhibit urate crystal-induced inflammation in mice. Ann Rheum Dis. 2015;74(3):587–594. 24326007 10.1136/annrheumdis-2013-203803

[27] Qiu L, Cheng XQ, Wu J, et al. Prevalence of hyperuricemia and its related risk factors in healthy adults from Northern and northeastern chinese provinces. BMC Public Health. 2013;13(1):664. 23866159 10.1186/1471-2458-13-664 PMC3722003

[28] Gao NN, Yu Y, Zhang BC, et al. Dyslipidemia in rural areas of North China: prevalence, characteristics, and predictive value. Lipids Health Dis. 2016;15(1):154. 27619340 10.1186/s12944-016-0328-y PMC5020547

[29] Kuwabara M, Niwa K, Hisatome I, et al. Asymptomatic hyperuricemia without comorbidities predicts cardiometabolic diseases Five-Year japanese cohort study. Hypertension. 2017;69(6):1036–1044. 28396536 10.1161/HYPERTENSIONAHA.116.08998 PMC5426964

[30] Wang WJ, Ren H, Tian QY, et al. Effects of occupational stress on blood lipids, blood sugar and immune function of doctors. Iran J Public Health. 2019;48(5):825–833. 31523638 PMC6717409

[31] Shankar A, Klein R, Klein BEK, et al. The association between serum uric acid level and long-term incidence of hypertension: population-based cohort study. J Hum Hypertens. 2006;20(12):937–945. 17024135 10.1038/sj.jhh.1002095

[32] Hak AE, Choi HK. Menopause, postmenopausal hormone use and serum uric acid levels in US women - The third national health and nutrition examination survey. Arthritis Res Ther. 2008;10(5):R116. 18822120 10.1186/ar2519 PMC2592803

[33] Han TS, Meng X, Shan RQ, et al. Temporal relationship between hyperuricemia and obesity, and its association with future risk of type 2 diabetes. Int J Obes (Lond). 2018;42(7):1336–1344. 29717279 10.1038/s41366-018-0074-5

[34] Engin A. Adiponectin-Resistance in obesity. Adv Exp Med Biol. 2017;960:415–441. 28585210 10.1007/978-3-319-48382-5_18

[35] Akande TO, Adeleye JO, Kadiri S. Insulin resistance in nigerians with essential hypertension. Afr Health Sci. 2013;13(3):655–660. 24250303 10.4314/ahs.v13i3.19 PMC3824445

[36] Shulman GI. Ectopic fat in insulin resistance, dyslipidemia, and cardiometabolic disease. N Engl J Med. 2014;371(23):2237–2238. 25470706 10.1056/NEJMc1412427

[37] Nakamura K, Sakurai M, Miura K, et al. HOMA-IR and the risk of hyperuricemia: a prospective study in non-diabetic japanese men. Diabetes Res Clin Pract. 2014;106(1):154–160. 25112919 10.1016/j.diabres.2014.07.006

[38] Zhao YT, Liu XT, Mao ZX, et al. Relationship between multiple healthy lifestyles and serum lipids among adults in rural China: a population-based cross-sectional study. Prev Med. 2020;138(6):106158. 32473269 10.1016/j.ypmed.2020.106158

[39] Aihemaitijiang S, Zhang YQ, Zhang L, et al. The association between Purine-Rich food intake and hyperuricemia: a Cross-Sectional study in chinese adult residents. Nutrients. 2020;12(12):3835. 10.3390/nu12123835 PMC7765492 33334038

